# Results of COVID-19 Surveillance in a Large United States Pediatric Healthcare System over One Year

**DOI:** 10.3390/children8090752

**Published:** 2021-08-30

**Authors:** Sarah E. Messiah, Luyu Xie, Matthew S. Mathew, George L. Delclos, Harold W. Kohl, Jeffrey S. Kahn

**Affiliations:** 1 University of Texas Health Science Center at Houston, School of Public Health, Dallas Campus, Dallas, TX 75390, USA; Luyu.Xie@uth.tmc.edu (L.X.); Matthew.S.Mathew@uth.tmc.edu (M.S.M.); 2 Center for Pediatric Population Health, UTHealth School of Public Health at Houston and Children’s Health System of Texas, Dallas, TX 75390, USA; 3 University of Texas Health Science Center at Houston, School of Public Health, Houston Campus, Houston, TX 77225, USA; George.Delclos@uth.tmc.edu; 4 University of Texas Health Science Center at Houston, School of Public Health, Austin Campus, Austin, TX, USA; Harold.W.Kohl@uth.tmc.edu; 5 Department of Kinesiology and Health Education, University of Texas at Austin, Austin, TX 78712, USA; 6 Department of Pediatrics, University of Texas Southwestern Medical Center, Dallas, TX 75390, USA; Jeffrey.Kahn@utsouthwestern.edu

**Keywords:** COVID-19, SARS-CoV-2, risk factors, health disparities, surveillance

## Abstract

Background: The lack of SARS-CoV-2 antigen surveillance testing in the pediatric population has inhibited accurate infection and hospitalization prevalence estimates. We aim to report the estimated prevalence of and risk factors for COVID-19 infection, hospitalization, and intensive care unit (ICU) admission across the three United States (US) waves in one of the largest pediatric healthcare systems in the nation. Methods: Retrospective electronic health record (EHR) review of all COVID-19 surveillance data among children aged 0–19 years seeking healthcare at one pediatric healthcare system that serves predominantly Medicaid-dependent families from 1 March 2020 to 31 March 2021. COVID-19 infection status (Y/N), hospital admission (Y/N), and ICU admission (Y/N) are the main outcomes. Results: Of 22,377 children aged ≤ 19 years tested for SARS-CoV-2 infection from March 2020–March 2021, 3126 were positive (14.0%), and out of those positive, 53.7% were hospitalized and 2.9% were admitted to the ICU. Compared to Wave 1 (1 March 2020–31 May 2020), the risk of a positive test increased from 16% (RR 1.16, 95% CI, 1.07–1.26) in Wave 2 (1 June 2020–31 October 2020) to 33% (RR 1.33, 95% CI, 1.23–1.44) in Wave 3 (1 November 2020–31 March 2021). Similarly, compared to Wave 1, the risk for hospitalization increased 86% (RR 1.86, 95% CI, 1.86–2.06) in Wave 2 and 89% in Wave 3 (RR 1.89, 95% CI, 1.70–2.08), and the risk for ICU admission increased from 10% in Wave 2 (RR 1.10, 95% CI, 0.39–3.01) to 310% in Wave 3 (RR 3.10, 95% CI, 1.21–7.80). Children with asthma, depressive disorders, type 1 or 2 diabetes, and anemia were more likely to be hospitalized while children with diabetes, obesity, cardiac malformations, and hypertension were more likely to be admitted to the ICU versus children without these conditions. Conclusions: Children were cumulatively impacted by the COVID-19 pandemic through the three US waves with more than a third hospitalized in Wave 3. Children with underlying health conditions were particularly at risk for severe illness and should be monitored for any long-term impacts.

## 1. Introduction

As of 17 June 2021 more than 4 million total pediatric COVID-19 cases, caused by the severe acute respiratory syndrome coronavirus 2 (SARS-CoV-2) infection, have been reported in the United States (US), representing 14.2% of cumulative cases [1]. However, this number is likely an underestimate due to a high proportion of mild and asymptomatic cases in the pediatric population and that testing was not widely available for children [2].

Of the more than 600,000 Americans who have died since the beginning of the pandemic, fewer than 450 have been under the age of 18 [1]. Pediatric COVID-19 hospitalizations are significantly lower than those for adults, further suggesting that children have less severe symptoms and illness from COVID-19 compared to adults [3]. However, of hospitalized children, one-third are admitted to intensive care where approximately six percent receive mechanical ventilation, and a small number develop multisystem inflammatory syndrome in children (MIS-C) that includes hypotension, severe abdominal pain, and cardiac dysfunction [4].

Reports by geographic locations indicate that pediatric COVID-19 cases are substantially greater in economically disadvantaged census tracts [5,6,7]. One of the main challenges, however, has been the absence of a denominator to estimate true incidence of COVID-19 in the pediatric population, in either community or healthcare based settings. Specifically, to date no pediatric COVID-19 surveillance data has been reported from large healthcare systems serving low resource patients and their families. Moreover, although several reports have documented clinical presentation of children with MIS-C [8], no reports to date have captured the proportion of children of all ages who tested positive stratified by hospitalization and intensive care unit (ICU) status through the third wave of the pandemic in the US. Here we report the trends of COVID-19 results and hospitalizations during a 13-month period in the 6th largest pediatric healthcare system in the country. It was hypothesized that pediatric cases would parallel similar trends as those seen in the US adult population.

## 2. Methods

### 2.1. Study Design

The UTHealth Committee for the Protection of Human Subjects reviewed and approved a retrospective cross-sectional electronic health record (EHR) (EPIC, Verona, WI, USA) analysis of pediatric patient records from 1 March 2020 to 31 March 2021. Office visit and hospital encounter data of all patients who had a COVID-19 test were extracted from the EHR including demographics, diagnoses, vital measures, body mass index (BMI), hospital and ICU admission status, hospital and ICU stay length, and outcomes.

### 2.2. Study Setting

Children’s Health System of Texas (CHST; serving 68% Medicaid and 60% ethnic minority families) is located in Dallas, Texas, which was a COVID-19 hot spot [9] throughout late winter 2021 [10]. The Centers for Disease Control and Prevention (CDC) defines a hotspot county as meeting all four of the following criteria, relative to the date assessed: (1) >100 new COVID-19 cases in the most recent 7 days, (2) an increase in the most recent 7-day COVID-19 incidence over the preceding 7-day incidence, (3) a decrease of <60% or an increase in the most recent 3-day COVID-19 incidence over the preceding 3-day incidence, and (4) the ratio of 7-day incidence/30-day incidence exceeds 0.31. In addition, hotspots must have met at least one of the following criteria: (1) >60% change in the most recent 3-day COVID-19 incidence, or (2) >60% change in the most recent 7-day incidence [10].

### 2.3. Study Participants

Participants included any child who aged 0–19 years presenting for medical care at CHST’s two main hospitals or ambulatory clinics for any reason from 1 March 2020 to 31 March 2021. All children, regardless of symptoms of COVID-19 were tested.

### 2.4. Exposures of Interest

Primary exposure of interest was SARS-CoV-2 across waves, typically defined by Texas-specific regional infectious patterns [10]. Secondary exposures included underlying medical conditions.

### 2.5. Primary Outcomes

**Figure 1:**
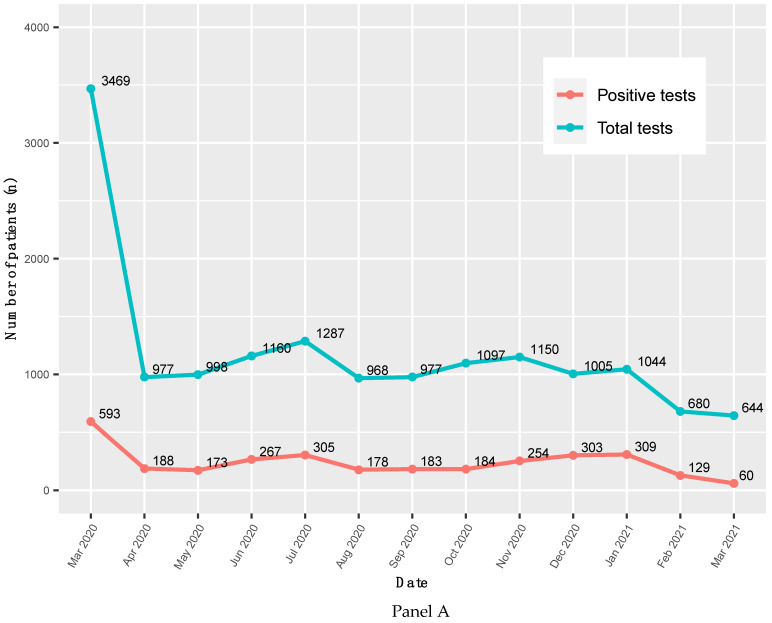

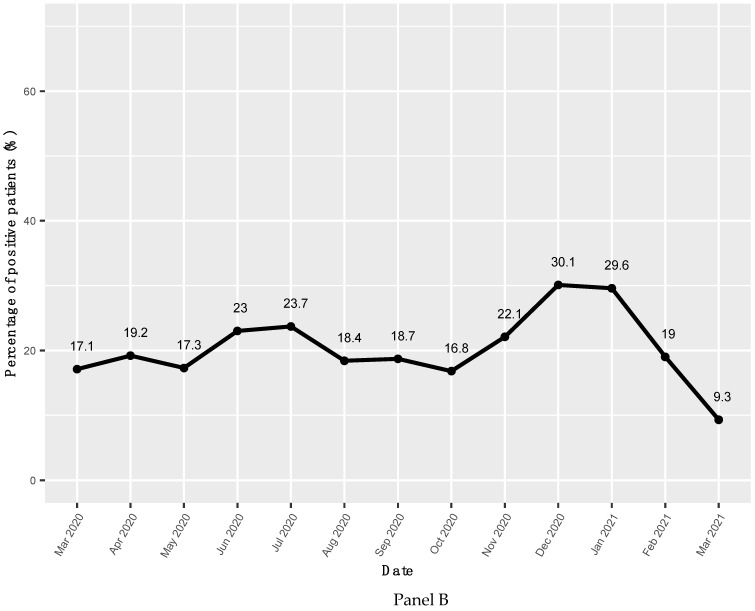
**Panel A**. The absolute number of COVID-19 tests results, March 2020–March 2021. *p*-trend for total tests = 0.042; *p*-trend for positive tests = 0.092. **Panel B**. Percentage of patients tested positive for COVID-19, March 2020–March 2021. *p*-trend = 0.834.

The three main outcomes of interest were: (1) a clinical diagnosis of COVID-19 (ICD-10 code of U07.1) confirmed with a positive COVID-19 test; (2) rate of hospitalization due to COVID-19; and (3) rate of ICU admissions due to COVID-19 by Wave 1 (1 March–31 May 2020), Wave 2 (1 June 2020–31 October 2020), and Wave 3 (1 November 2020–31 March 2021). Waves are typically determined based on the infectious pattern in the local region [9,10]; therefore, the 3 waves [11] were determined by locally driven COVID-19 infection patterns (Figure 1, Panel B). COVID-19 infection status was confirmed using a nasopharyngeal swab specimen with either rapid antigen test (RAT) or real-time reverse transcription polymerase chain reaction (RT-qPCR) test.

### 2.6. Measures

All patient comorbidities were extracted from the International Classification of Diseases, Tenth Revision, Clinical Modification (ICD-10-CM) diagnosis codes, and included asthma, anemia, anxiety, cardiac malformation, depressive disorders, diabetes (type 1 and type 2), epilepsy, hypertension, neurodevelopmental disorders, obesity, substance use disorders, and injury/trauma (ICD-10 codes are listed in Supplemental Table S1). Similarly, COVID-19 related common symptoms [12] were also determined using ICD-10 codes, including fever (R50.9), cough (R05), nasal congestion (R09.81), shortness of breath (R06.02), diarrhea (R19.7), headache (R51, R51.9), nausea or vomiting (R11.10, R11.11, R11.2), and loss of taste or smell (R43, R43.2, R43.8, R43.9), chills without fever (R68.83), pain/sore throat (R07.0, J02.9), fatigue (R53.83), and muscle pain (R52). A patient could report more than one symptom.

### 2.7. Covariates

Covariates of interests include age, sex, race/ethnicity, BMI, insurance, and comorbidities.

## 3. Statistical Analysis

Categorical variables were presented as frequencies and percentages, and continuous variables were summarized as means (standard deviation [SD]). The demographic and baseline characteristics of pediatric patients with COVID-19 positive results were compared by hospital admission (Y/N) and ICU admission (Y/N) status by Pearson Chi-square tests, Fisher’s exact tests or two-sample t-test with equal or unequal variance, respectively. Age and BMI were analyzed as both continuous variable and categorical variable with three levels. A CDC SAS macro program was used to calculate BMI percentiles based on US national growth charts and obesity was defined as ≥95th%ile adjusted for age and sex [13]. COVID-19 related symptoms were compared by COVID test results (positive vs. negative) via Pearson Chi-square tests. Generalized linear models with normal distribution and identity link function were built to assess time trend of COVID-19 test results by the total of number tests and percentage of positive results. By using patients from Wave 1 as the reference group, relative risks of Wave 2 and Wave 3 for positive tests, hospital admission, and ICU admission were computed by the following equation, respectively:

$$\mathrm{Relative}\;\mathrm{Risk}=\frac{\mathrm{Incidence}\;\mathrm{rate}\;\mathrm{in}\;\mathrm{Wave}\;2\;\mathrm{or}\;\mathrm{Wave}\;3}{\mathrm{Incidence}\;\mathrm{rate}\;\mathrm{in}\;\mathrm{Wave}\;1}$$

Three stepwise logistic regression models using *p*-value < 0.1 and < 0.05 as entrance and retention criteria were created to explore possible predictors of a positive test (Y/N), hospital admission (Y/N), and ICU admission (Y/N), separately. Independent variables included age, sex, race/ethnicity, BMI, and comorbidities (Y/N), such as asthma, anemia, anxiety, cardiac malformation, depressive disorders, diabetes (type 1 and type 2), epilepsy, hypertension, neurodevelopmental disorders, obesity, substance use disorders, and injury/trauma. All statistical analyses were performed using SAS v9.4 (SAS Institute, Cary, NC, USA) and R v4.0.2 (R core team, 2020). Two-sided *p*-value < 0.05 is considered significant.

## 4. Results

Between 1 March 2020 and 31 Mach 2021, a total of 23,719 CHST unique patients had a COVID-19 PCR test. After excluding those aged greater than 19 years old (n = 1342), the analytical sample included 22,377 unique patients. Out of those, the analytical sample used for analysis here included 3126 (14.0%) children with a positive test. Among those with a positive test, 1679 (53.7%) children were hospitalized and 49 (1.6%) were admitted to the ICU. (Supplemental Figure S1). The absolute number of children with a positive test ranged from 593 in March 2020 to 60 in March 2021 (Figure 1, Panel A). Over time, the prevalence of positive tests was 17.1% in March 2020, peaked at 30.1% in December 2020, then fell to 9.3% in March 2021. (Figure 1, Panel B).

**Figure 2:**
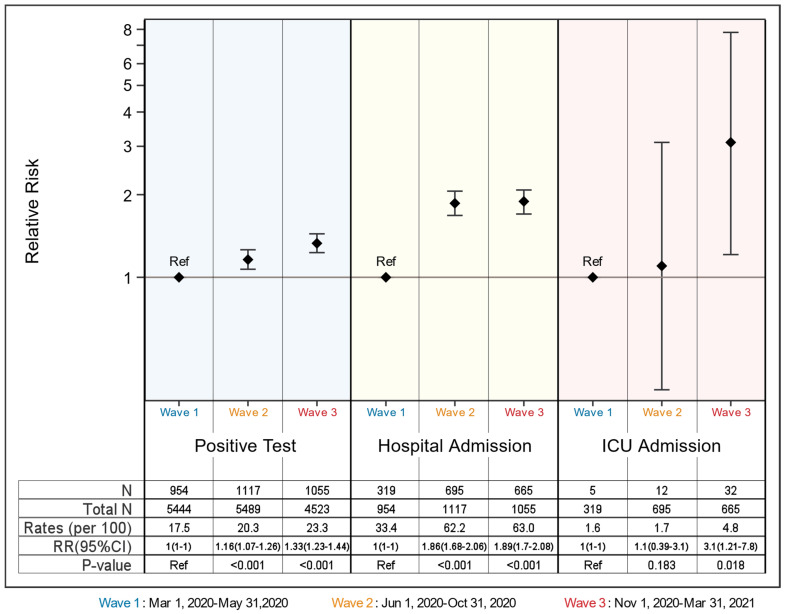
The relative risk of Wave 1 versus Wave 2 and Wave 3 by positive COVID-19 tests results, hospital admission, and ICU admission.

Patients in Wave 3 and Wave 2 had 1.33 (95% 1.07–1.26) and 1.16 (95% 1.23–1.44) times higher risk of having a positive test, respectively. (Figure 2) Similarly, the risk of hospitalization increased by ~80% in Wave 2 and 3 (RR_wave 2_ = 1.86, 95% CI 1.68–2.06; RR_wave 3_ = 1.89, 95% CI 1.70–2.08) versus Wave 1. Approximately 5 out of every 100 patients were admitted to the ICU in Wave 3 while 1.6 out of 100 were admitted to ICU in Wave 1 (expressed as a relative risk of 3.1; 95% CI 1.21–7.8). Overall, children in Wave 3 had more than a 3 times higher risk of ICU admission versus children in Wave 1 and 2.

The mean (SD) age of hospitalized children was 8.0 (6.1) years (38.4% aged 0–5 years, 27.2% aged 6–12 years and 31.8% aged 13–19 years) across all three waves. The majority (51.7%, n = 868) of hospitalized children were male and 67.8% (n = 1138) were non-Hispanic White followed by non-Hispanic Black (18.1%, n = 303), other/multi-race (10.8%, n = 181), and Hispanic (3.4%, n = 57). Most (95.3%) hospitalized children had a normal BMI (BMI percentile, mean [SD], 68.3 [32.0]). There was no difference in hospitalized and non-hospitalized children’s age (8.4 years, SD 6.0, 38.4% aged 0–5 years, 28.7% aged 6–12 years, and 32.9% aged 13–19 years, respectively), sex (45.3% female, 54.6% male), race/ethnicity (66.3% non-Hispanic White, 17.5% non-Hispanic Black, 2.8% Hispanic), or raw BMI (mean 22.0 kg/m^2^, SD 9.5). However, significantly more hospitalized patients were covered by government insurance than non-hospitalized patients (77.5% versus 72.8%, *p* < 0.001). Comorbidities were more common in hospitalized versus non-hospitalized children, particularly asthma (4.4% vs. 1.8%), depressive disorders (2.0% vs. 0.4%), diabetes (1.6% vs. 0.8%), and anemia (1.2% vs. 0.3%). The mean length of hospital stay was 17.4 h (SD 54.4). Other vital signs stratified by age group are included in Table 1.

The mean age (SD) for non-ICU children was significantly younger than ICU children (8.0 (6.1) versus 10.0 (6.0), *p* = 0.022). There was no difference in sex, race/ethnicity, and insurance types. Notably, significantly more patients with BMI ≥ 95th percentile were admitted to the ICU (18.4% vs. 4.3%, *p* < 0.001). Similarly, compared to non-ICU patients, ICU children had a higher prevalence of other conditions including asthma (8.2% vs. 4.3%), neurodevelopmental disorders (10.2% vs. 2.0%), diabetes (20.4% vs. 0.1%), and cardiac malformation (10.2% vs. 0.4%). The mean hospital length of stay for ICU patients was 166.9 h (SD 157.3), and on average 69.2 (SD 85.1) hours were spent in the ICU. The hospital stay for non-ICU patients was significantly shorter (mean 12.9 h, SD 40.4, *p* < 0.001). Younger children admitted to ICU had significantly lower blood pressure (mean (SD), 92.7 (11.2)/54.6(6.7) vs. 101.1 (10.6)/64.8(10.2) mmHg), and pulse oximetry (mean (SD), 94.8(3.4) vs. 97.8 (1.3)), temperature (mean (SD), 98.1 (0.7) vs. 98.9 (1.0)), and higher respiration (mean (SD), 37.1 (8.4) vs. 29.7 (5.6)) versus non-admitted patients (Table 1).

Children with a positive COVID-19 test were more likely to report a symptom than those with a negative test (29.8% vs. 18.7%, *p* < 0.001). However, it is notable that the majority (70.2%) of children without any common symptoms had a positive test. Nasal congestion was the only symptom that did not differ between positive and negative groups (2.9% vs. 2.4%, *p* = 0.078) (Table 2).

After controlling for potential confounders, significant risk factors for positive COVID-19 infection include older age (6–12 years versus 0–5 years: adjusted odds ratio (aOR) 1.31, 95% confidence interval (CI) 1.18–1.44; 13–19 years versus 0–5 years: aOR 2.27, 95% CI 2.04–2.53), obesity (BMI percentile ≥ 95%th, aOR 1.30, 95% CI 1.10–1.54), and a diagnosis of a depressive disorder (aOR 1.60, 95% CI 1.05–2.43), or diabetes (aOR 1.62, 95% 1.07–2.44) (Table 3). Non-Hispanic Black children (aOR 0.68, 95% CI 0.61–0.75) and children covered by private insurance (aOR 0.69, 95% CI 0.62–0.76) were less likely to test positive than their ethnic group counterparts. Children with asthma had lower odds of a positive test (aOR 0.62, 95% CI 0.50–0.78) versus children without asthma. Children with obesity had 60% lower odds for hospital admission (aOR 0.40, 95% CI 0.29–0.53), while children with depressive disorders (aOR5.68, 95% CI 2.18–14.78), anemia (aOR 4.46, 95% CI 1.51–13.16), asthma (aOR 2.75, 95% CI 1.70–4.43), or diabetes (aOR 2.12, 95% CI 1.02–4.43) had a significantly higher odds of hospital admission.

Children with obesity had more than four times higher odds of ICU admission (aOR 4.6, 95% CI 1.9–10.8). Comorbidities dramatically increased the odds of ICU admission. Specifically, children with diabetes (aOR 25.72, 95% CI 10.49–63.06), cardiac malformation (aOR 46.69, 95% CI 13.5–161.9), or hypertension (aOR 11.21, 95% CI 1.27–99.04) had 11 to 46 times higher odds of ICU admission, respectively, compared to their counterparts (Table 3).

## 5. Discussion

This analysis shows the proportion of children of all ages who tested positive for COVID-19 stratified by hospitalization and intensive care unit (ICU) status through the US third wave of the pandemic. In this analysis over 13 months in one large pediatric healthcare system serving predominantly low-income families, among the over 22,000 who received a COVID-19 test, results showed that the risk of a positive COVID-19 antigen test, hospitalization, and ICU admission increased over time with each subsequent wave. The majority (70%) of those with a positive test had no symptoms. Comorbidities including type 1 or type 2 diabetes, hypertension, obesity, and cardiac malformations increased the odds of ICU admission significantly, while children with asthma, depressive disorders, or diabetes were significantly more likely to be hospitalized. Collectively these findings show that the third wave of the US COVID-19 pandemic had a significant impact on children from low resource backgrounds and with underlying health conditions in particular.

Analysis of CDC COVID-NET data showed that in the first and second wave of the pandemic (March 1–25 July 2020) the cumulative COVID-19-associated hospitalization rate among children aged <18 years was 8.0 per 100,000 population, with the highest rate among children aged <2 years [14]. Reports from China’s CDC corroborate those of US findings in that children were less susceptible to hospitalization including before COVID-19 hit this country (16 January 2020 to 8 February 2020) [15]. Systematic reviews that included studies from the US, Italy, and the UK among others during the first 6 months of 2020 also supported this finding [16,17]. There are few [18], if any, data on how the third and most deadly wave of the pandemic has impacted the pediatric population in all age groups. COVID-NET analysis of all three US waves among 12–17 year olds only showed that 31.4% were admitted to an intensive care unit (ICU) [18], similar to our results here among all age groups. Results here suggest that it was more consequential in terms of not only infections among children, but severe illness resulting in ICU admission versus the first wave for all age groups. 

Results from the CDC COVID-NET analysis also showed that Hispanic and non-Hispanic Black children had higher cumulative rates of COVID-19-associated hospitalizations (16.4 and 10.5 per 100,000, respectively) versus non-Hispanic White children (2.1 per 100,000) [14]. In agreement with the COVID-NET studies, another study of 33 healthcare organizations showed the risk of hospitalization was greater in non-Hispanic Black and Hispanic children compared with non-Hispanic White children [19]. However, our results showed that non-Hispanic Black children were 32% less likely to be hospitalized versus non-Hispanic Whites and Hispanic and mixed race/other children were also slightly less likely to be hospitalized. Another analysis using the CDC data show that hospitalization and in-hospital death were rare in children diagnosed with COVID-19 but children at higher risk for these outcomes include those with an underlying medical condition, as well as non-Hispanic Black children [20]. We excluded children who died from this analysis. A more recent analysis through Wave 2 and partially into Wave 3 (March 2020 through January 2021) using the COVID-NET data found a higher risk of severe COVID-19 illness among children with medical complexity and certain underlying conditions, such as type 1 diabetes, cardiac and circulatory congenital anomalies, and obesity [21]. These results are similar to those found here in that children with either type 1 or type 2 diabetes were at increased risk for both hospitalization and ICU admissions [21].

Results here showed that children with obesity had increased odds of a positive COVID-19 test and IC admission, but not hospitalization. This finding may be due to children with obesity presenting with more severe illness requiring immediate ICU admission. Obesity is a known risk factor for severe COVID-19 illness in adults [21] and especially severe obesity [22] but not necessarily a COVID-19 diagnosis. Our results are also consistent with other studies that show that children with asthma, diabetes, and mental health disorders including depression are at increased risk for hospitalization [21,23]. Conversely, children with asthma in particular were less likely to have a positive COVID diagnosis, but more likely to be hospitalized, which is predictable given the pulmonary nature of the disease, but not in agreement with other national studies [18].

More than 70% of children with a COVID-19 positive diagnoses did not report any common symptoms suggested by CDC [12]. This is in agreement with other studies both in the US and globally [2,24]. This finding suggests that the virus had been circulating widely, and silently in the majority of children in the geographic catchment area that was a COVID-19 hot spot [9].

## 6. Limitations

This study had limitations. First, the use of ICD-10-CM diagnostic codes to identify COVID-19 cases might have resulted in misclassification bias, although there were few other cases of similar respiratory illness (e.g., influenza, RSV) during the same time period. Specifically, a COVID-19 specific ICD-10 code was not available until January 2021 [25] and thus may have not accurately captured children with a previous diagnosis. Second, the use of ICD-10-CM codes to identify underlying medical conditions may have also resulted in misclassification bias. Indeed, others have suggested that miscoding of medical claims can lead to misclassification bias from ICD codes [26]. Third, due to the cross-sectional study design and analysis, casual relationships cannot be inferred between previously existing medical conditions and diagnoses and severe COVID-19 illness. Fourth, because EHR data were used to retrospectively capture hospitalization and ICU admission status, the temporality may be affected. However, the pattern of hospitalization data in the present analysis is similar to the CDC’s report indicting the accuracy of our results [24]. Fifth, the analysis only included frequent chronic conditions, and thus rare conditions were not assessed as potential risk factors for hospitalization and ICU admission, and thus the results presented here are not representative of all children with COVID-19. Finally, the characteristics of the pediatric population who had physical visits may be different from those who completed telehealth visits.

## 7. Conclusions

This analysis can be summarized in the following three conclusions: (1) the proportion of children of all ages who tested positive for SARS-CoV-2 stratified by hospitalization and ICU status increased across three waves; (2) children with diabetes, hypertension, obesity, and cardiac malformations were particularly at risk for ICU admission in the third wave; and (3) collectively these findings show that the third wave of the US COVID-19 pandemic had a significant impact on children with underlying health conditions and from low resource backgrounds and was more severe than earlier waves.

## Figures and Tables

**Table 1:** Demographics and baseline characteristics of pediatric patients with COVID-19 positive results by hospitalization and intensive care unit (ICU) admission status, March 2020–March 2021.

	Hospital Admission Status			ICU Admission Status		
	Hospitalization (n = 1679)	No Hospitalization (n = 1447)	*p*-Value ^a^	ICU (n = 49)	No ICU (n = 1630)	*p*-Value ^b^
**Age, mean (SD), years**	8.0 (6.1)	8.4 (6.0)	0.075	10.0 (6.0)	8.0 (6.1)	0.022
0–5 years, n (%)	688 (38.4)	556 (38.4)	0.341	13 (26.5)	675 (41.4)	0.067
6–12 years, n (%)	457 (27.2)	415 (28.7)		14 (28.6)	443 (27.2)	
13–19 years, n (%)	534 (31.8)	476 (32.9)		22 (44.9)	512 (31.4)	
**Sex, n (%)**			0.079			0.100
Male	868 (51.7)	790 (54.6)		31 (63.3)	837 (51.4)	
Female	811 (48.3)	655 (45.3)		18 (36.7)	793 (48.6)	
**Race/ethnicity, n (%)**			0.119			0.529
Caucasian	1138 (67.8)	960 (66.3)		29 (59.2)	1109 (68.0)	
African American	303 (18.1)	253 (17.5)		10 (20.4)	293 (18.0)	
Hispanic	57 (3.4)	40 (2.8)		2 (4.1)	55 (3.4)	
Other/Multi-race	181 (10.8)	194 (13.4)		8 (16.3)	173 (10.6)	
**Body mass index percentile, mean (SD)**	68.3 (32.0)	70.7 (31.8)	0.346	66.7 (7.2)	68.5 (31.7)	0.799
<95th percentile, n (%)	1600 (95.3)	1289 (89.1)	<0.001	40 (81.6)	1560 (95.7)	<0.001
≥95th percentile, n (%)	79 (4.7)	158 (10.9)	<0.001	9 (18.4)	70 (4.3)	0.061
**Insurance types, n (%)**						
Government	1302 (77.5)	966 (72.8)		32 (65.3)	1270 (77.9)	
Private	300 (17.9)	297 (22.4)		15 (30.6)	285 (17.5)	
Self-pay/Other	77 (4.6)	63 (4.8)		2 (4.1)	75 (4.6)	
**Known COVID exposure, n (%)**	148 (8.8)	117 (8.1)	0.479	6 (12.4)	142 (8.7)	0.437
**Comorbidities, n (%)**						
Injury/trauma	76 (4.5)	58 (4.0)	0.536	1 (2.0)	75 (4.6)	0.723
Asthma	74 (4.4)	26 (1.8)	<0.001	4 (8.2)	70 (4.3)	0.166
Neurodevelopmental disorders	38 (2.3)	29 (2.0)	0.711	5 (10.2)	33 (2.0)	0.004
Depressive disorders	33 (2.0)	5 (0.4)	<0.001	2 (4.1)	31 (1.9)	0.250
Neoplasms	31 (1.8)	18 (1.2)	0.176	1 (2.1)	30 (1.8)	0.918
Any diabetes	26 (1.6)	11 (0.8)	0.047	10 (20.4)	16 (0.1)	<0.001
Type 1 diabetes	0	0	-	0	0	-
Type 2 diabetes	9 (0.5)	4 (0.3)	0.404	1 (2.0)	8 (0.5)	0.235
Obesity/overweight	20 (1.2)	15 (1.0)	0.735	1 (2.0)	19 (1.2)	0.449
Anemia	20 (1.2)	4 (0.3)	0.003	1 (2.0)	19 (1.2)	0.449
Anxiety	17 (1.0)	11 (0.8)	0.569	0 (0)	17 (1.0)	1.0
Epilepsy	13 (0.8)	24 (1.7)	0.030	1 (2.0)	12 (0.7)	0.321
Cardiac malformation	11 (0.7)	17 (1.2)	0.132	5 (10.2)	6 (0.4)	<0.001
Hypertension	6 (0.4)	2 (0.1)	0.299	1 (2.0)	5 (0.3)	0.163
Substance use disorders	4 (0.2)	1 (0.1)	0.382	4 (0.3)	0 (0)	1.0
**Hospital length of stay, mean (SD), hours**	17.4 (54.4)	N/A	N/A	166.9 (157.3)	12.9 (40.4)	<0.001
**ICU length of stay, mean (SD), hours**	N/A	N/A	N/A	69.2 (85.1)	N/A	N/A
**Vital signs, mean (SD) ^c^**						
**Systolic blood pressure**						
0–5 years	100.9 (10.7)	N/A	N/A	92.7 (11.2)	101.1 (10.6)	0.005
6–12 years	110.0 (9.1)	N/A	N/A	107.5 (7.7)	110.1 (9.1)	0.307
13–19 years	118.4 (10.1)	N/A	N/A	114.9 (11.9)	118.6 (9.9)	0.093
**Diastolic blood pressure**						
0–5 years	64.6 (10.2)	N/A	N/A	54.6 (6.7)	64.8 (10.2)	<0.001
6–12 years	68.8 (8.1)	N/A	N/A	67.2 (8.0)	68.9 (8.2)	0.435
13–19 years	72.8 (8.2)	N/A	N/A	65.5 (8.3)	72.9 (8.1)	0.057
**Pulse**						
0–5 years	134.9 (20.1)	N/A	N/A	138.2 (17.6)	134.9 (20.1)	0.552
6–12 years	101.0 (16.1)	N/A	N/A	105.1 (16.8)	100.9 (16.1)	0.330
13–19 years	88.4 (15.1)	N/A	N/A	85.1 (17.9)	88.6 (14.9)	0.287
**Pulse oximetry**						
0–5 years	97.7 (1.4)	N/A	N/A	94.8 (3.4)	97.8 (1.3)	0.010
6–12 years	97.5 (1.5)	N/A	N/A	97.1 (1.0)	97.5 (1.5)	0.288
13–19 years	97.6 (1.4)	N/A	N/A	96.3 (1.9)	97.6 (1.3)	0.005
**Respiration**						
0–5 years	29.9 (5.8)	N/A	N/A	37.1 (8.4)	29.7 (5.6)	0.008
6–12 years	21.9 (3.4)	N/A	N/A	25.1 (7.7)	21.8 (3.1)	0.130
13–19 years	19.3 (2.4)	N/A	N/A	20.3 (5.4)	19.3 (2.1)	0.379
**Temperature**						
0–5 years	98.8 (1.0)	N/A	N/A	98.1 (0.7)	98.9 (1.0)	0.003
6–12 years	98.5 (0.9)	N/A	N/A	98.2 (0.5)	98.6 (0.9)	0.023
13–19 years	98.4 (1.8)	N/A	N/A	98.2 (0.5)	98.4 (1.8)	0.058

^a^ Comparison of patients admitted to hospital vs. patients not admitted to hospital using Pearson chi-square test, Fisher’s exact test, or two-sample *t*-test with equal or unequal variance. ^b^ Comparison of patients admitted to ICU vs. patients not admitted to ICU using Pearson chi-square test, Fisher’s exact test, or two sample *t*-test with equal or unequal variance. ^c^ Vital signs for non-hospitalized patients from community primary care clinics were not available for our study.

**Table 2:** COVID-19 test results by presenting symptoms.

Symptoms	COVID Test Positive (n = 3126)	COVID Test Negative (n = 19,251)	*p*-Value ^a^
**Fever, n (%)**			<0.001
Yes	559 (17.9)	2161 (11.2)	
No	2567 (82.1)	17,090 (88.8)	
**Cough, n (%)**			<0.001
Yes	176 (5.6)	655 (3.4)	
No	2950 (94.4)	18,596 (96.6)	
**Nausea or vomiting, n (%)**			<0.001
Yes	162 (5.2)	697 (3.6)	
No	2964 (94.8)	18,554 (96.4)	
**Headache, n (%)**			<0.001
Yes	139 (4.5)	222 (1.2)	
No	2987 (95.5)	19,029 (98.8)	
**Pain/sore throat, n (%)**			<0.001
Yes	97 (3.1)	336 (1.8)	
No	3029 (96.9)	18,915 (98.2)	
**Diarrhea, n (%)**			<0.001
Yes	94 (3.0)	329 (1.7)	
No	3032 (96.7)	18,922 (98.3)	
**Nasal congestion, n (%)**			0.078
Yes	91 (2.9)	459 (2.4)	
No	3035 (97.1)	18,792 (97.6)	
**Shortness of breath, n (%)**			<0.001
Yes	42 (1.3)	88 (0.5)	
No	3084 (98.7)	19,163 (99.5)	
**Loss of taste or smell, n (%)**			<0.001
Yes	31 (1.0)	12 (0.1)	
No	3094 (99.0)	19,239 (99.9)	
**Fatigue, n (%)**			<0.001
Yes	23 (0.7)	52 (0.3)	
No	3103 (99.3)	19,199 (99.7)	
**Muscle pain, n (%)**			<0.001
Yes	14 (0.5)	28 (0.1)	
No	3112 (99.5)	19,223 (99.9)	
**Chills without fever, n (%)**			0.500
Yes	3 (0.1)	12 (0.06)	
No	3123 (99.9)	19,239 (99.94)	
**Any symptoms above, n (%)**			<0.001
Yes	933 (29.8)	3594 (18.7)	
No	2193 (70.2)	15,657 (81.3)	

^a^ Pearson chi-square test.

**Table 3:** Adjusted odds of positive COVID-19 test results, hospital and ICU admission by patient characteristics and comorbidities.

Variable	Positive COVID-19 Test ^a^		Hospital Admission ^b^		ICU Admission ^c^	
	aOR (95% CI)	*p*-Value	aOR (95% CI)	*p*-Value	aOR (95% CI)	*p*-Value
**Age**						
0–5 years (ref)	1.0	-	-	-	-	-
6–12 years	1.31 (1.18–1.44)	0.002	-	-	-	-
13–19 years	2.27 (2.04–2.53)	<0.001	-	-	-	-
**Race/Ethnicity**						
Caucasian (ref)	1.0	-	-	-	-	-
Non-Hispanic Black	0.68 (0.61–0.75)	<0.001	-	-	-	-
Hispanic	0.91 (0.71–1.16)	0.694	-	-	-	-
Other/Multi-race	0.95 (0.84–1.09)	0.137	-	-	-	-
**BMI percentile**						
<95%th (ref)	1.0	-	1.0	-	1.0	-
≥95%th	1.30 (1.10–1.54)	0.002	0.40 (0.29–0.53)	0.002	4.6 (1.9–10.8)	<0.001
**Insurance**						
Government (ref)	1.0	-	1.0	-	-	-
Private	0.69 (0.62–0.76)	<0.001	0.71 (0.69–0.85)	0.025	-	-
Self-pay/Other	1.06 (0.87–1.29)	0.015	0.87 (0.61–1.23)	0.857	-	-
**Asthma**						
No (ref)	1.0	-	1.0	-	-	-
Yes	0.62 (0.50–0.78)	<0.001	2.75 (1.70–4.43)	<0.001	-	-
**Depressive disorders**						
No (ref)	1.0	-	1.0	-	-	-
Yes	1.60 (1.05–2.43)	0.027	5.68 (2.18–14.78)	<0.001	-	-
**Any diabetes**						
No (ref)	1.0	-	1.0	-	1.0	-
Yes	1.62 (1.07–2.44)	0.023	2.12 (1.02–4.43)	0.045	25.72 (10.49–63.06)	<0.001
**Anemia**						
No (ref)	-	-	1.0	-	-	-
Yes	-	-	4.46 (1.51–13.16)	0.007	-	-
**Epilepsy**						
No (ref)	-	-	1.0	-	-	-
Yes	-	-	0.46 (0.23–0.94)	0.032	-	-
**Cardiac malformation**						
No (ref)	-	-	-	-	1.0	-
Yes	-	-	-	-	46.69 (13.5–161.9)	<0.001
**Hypertension**						
No (ref)	-	-	-	-	1.0	-
Yes	-	-	-	-	11.21 (1.27–99.04)	0.030

aOR, adjusted Odds Ratio. ^a^ Stepwise logistic regression model to find predictors for positive test results (versus negative results) adjusting for age, sex, race/ethnicity, BMI percentile, and comorbidities. ^b^ Stepwise logistic regression model to find predictors for hospital admission (versus no admission) adjusting for age, sex, race/ethnicity, BMI percentile, and comorbidities. ^c^ Stepwise logistic regression model to find predictors for ICU admission (versus no ICU admission) adjusting for age, sex, race/ethnicity, BMI percentile, and comorbidities.

## Data Availability

The data presented in this study are available on request from the corresponding author. The data are not publicly available due to ethical restrictions.

## Supplementary Materials

The following are available online at www.mdpi.com/XXX/s1, Figure S1: Flow chart of study participants, Table S1: ICD-10-CM codes for comorbidities.
