# Supplementary material for: Results of COVID-19 Surveillance in a Large United States Pediatric Healthcare System over One Year
Source: Children (Basel). 2021 Aug 30;8(9):752. doi: 10.3390/children8090752 (PMC8468442; doi:10.3390/children8090752)
Supplement: Supplementary file 1 [file children-08-00752-s001.zip › Supplemental Table 1.pdf]

Supplemental table 1. ICD-10-CM codes for comorbidities.

**Comorbidities**	**ICD-10-CM**
Asthma	J45
Anemia	D60-D64
Anxiety	F40- F48
Cardiac malformation	Q20-Q28
Depressive disorders	F32; F33
Epilepsy	G40
Hypertension	I10-I16
Neurodevelopmental disorders	F50-F99
Obesity	E65-E68
Substance use disorders	F10-F19
Type 1 diabetes	E10
Type 2 diabetes	E11
Any diabetes	E08-E13
Injury/Trauma	S00-S99; T07; T14
