# Supplementary figures and images for: Results of COVID-19 Surveillance in a Large United States Pediatric Healthcare System over One Year
Source: Children (Basel). 2021 Aug 30;8(9):752. doi: 10.3390/children8090752 (PMC8468442; doi:10.3390/children8090752)

**Supplemental figure 1.** Flow chart of study participants.

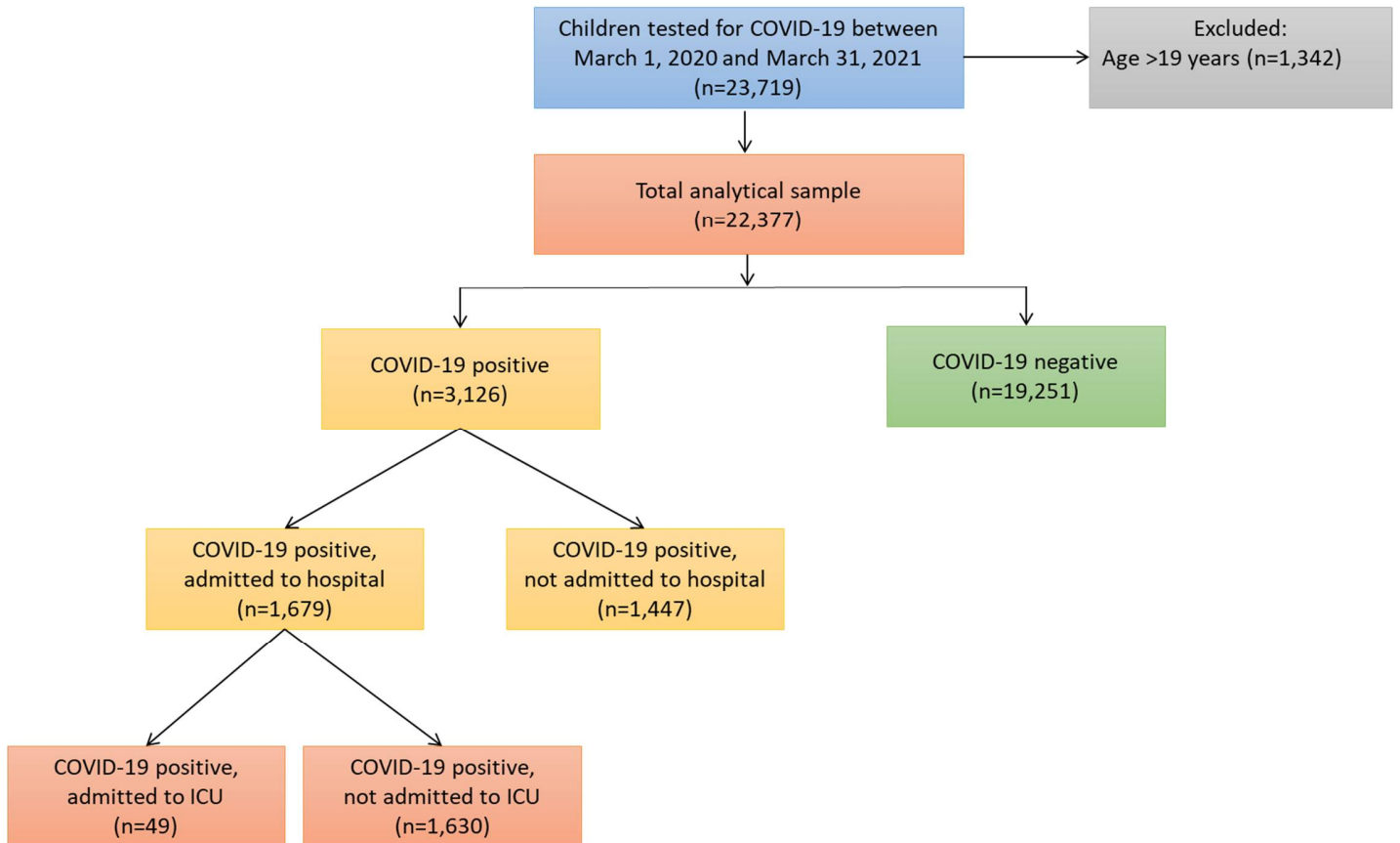

Supplement: Supplementary file 1 [file children-08-00752-s001.zip › Supplemental figure 1.pdf]
